# Supplementary material for: SyNDI: synchronous network data integration framework
Source: BMC Bioinformatics. 2018 Nov 6;19:403. doi: 10.1186/s12859-018-2426-5 (PMC6219086; doi:10.1186/s12859-018-2426-5)
Supplement: Supplementary file 5 — Files of the example presented in Additional file 4. This additional file is a zip package that contains all files used in Additional file 4. Detailed descriptions of each of these files are presented in a README file included in this zip package. (GZ 30411 kb) [file 12859_2018_2426_MOESM5_ESM.gz › SyNDITools/Meme2Fimo/meme_4.9.0_4.tar.gz!/meme_4.9.0/doc/release-notes.html]

MEME Suite - Release Notes


|  |  |
| --- | --- |
|  | MEME Suite The MEME Suite web application requires the use of JavaScript   Javascript doesn't seem to be available on your browser.  MEME Suite System Release Notes   ---   **MEME version 4.9.0 patch 4 -- January 29, 2013**   - **New Features and Enhancements**   - CentriMo     - Added computation of *p-*value ratio whenever       negative sequences are given. The *p-*value ratio       seems to be more useful than the discriminative       *p-*value computed in the `--disc` mode,       and is much faster to compute. Motifs are now sorted by       *p-*value ratio by default when negative sequences       are given and `--disc` is not.     - Added feature to supress plotting of negative sequence       enrichment curves so a single run can be used for       comparative and absolute enrichment analysis.- Sorting on Region Center now sorts by increasing         absolute value (centrality) of the location of the region.       - The "region" sorting feature menu is now locked to the         same value as the "motifs" feature menu, unless a checkbox         is checked. This helps prevent confusion.       - Improved documentation popups.       - Fixed bug in the Fisher Exact test (affected AME,         too).       - Added new standalone utility: fisher\_exact   - MEME-ChIP     - Added -centrimo-local switch to allow CentriMo to perform       local motif enrichment as part of MEME-ChIP.     - FIMO       - Added `--parse-genomic-coords` option.         This option turns on parsing of UCSC style genomic         coordinates from sequence headers.     - taipale2meme       - Add options [-nc ]+ and [-oc ] to accomodate         format of Jolma2013 Cell paper. - **Bug fixes**   - Fixed buffer overflow bug in shift\_sequence().   - Fixed bug in DREME: -norc option resulted in incorrect PSSM.  ---   **MEME version 4.9.0 -- October 2, 2012**   - **New Features and Enhancements**   - CentriMo     - Added local mode for detecting uncentered enriched       regions.     - Added discriminative mode which takes two datasets       and uses the Fisher exact test to detect discriminatively       enriched regions.     - Added listing of sequence IDs in output for best       enriched regions.     - Added score threshold optimization mode.     - Updated website form to give access to local mode and       discriminative mode.   - FIMO     - Simplified command line options controlling output threshold.     - FIMO no longer emits a wiggle format file.       The wiggle file contained the same information as the GFF file,       but was more verbose.     - Output of '--text' option now matches format of 'fimo.txt' file.     - Text file now provides explicit strand information.     - Text file now lists start and stop coordinates in increasing order relative to the positive strand       for all matches.     - HTML output is now limited to the 1000 most significant matches.   - MEME-ChIP     - Complete rewrite of the MEME-ChIP program to run MEME,       DREME, Tomtom and CentriMo.     - New output which collates all the motifs found by the       called programs with links to their outputs.     - New website form allows choice of motif database and       adds more options for DREME and CentriMo.   - TOMTOM     - Creation of EPS and PNG Logo files is now optional to       save disk space.       Two new switches (-png and -eps) have been added to the       command line version of TOMTOM.   - meme-rename     - Added new script meme-rename for mass renaming of       output html files so they can inhabit the same directory.   - psp-gen     - Added handling of ambiguous IUPAC alphabet       symbols for DNA bases.   - New Motif Databases     - Malaria transcription factor motifs from PBM experiments       (Campbell et al., 2010 PLos Pathogens)     - New motifs derived from the original UniPROBE PBM data using       a different algorithm (Zhao and Stormo,       2011 Nature Biotechnology) - **Bug fixes**   - Fixed FIMO bug--nucleotide motifs were labeled as polypeptide motifs in GFF output   - Fixed FIMO bug--both motifs and RC motifs were printing in list of best possible matches   - Fixed CentriMo bug--"--motif" option was not functional.- Fixed bug in gendb--was failing with higher-order models.- Fixed bug in gendb--was failing with higher-order models.- Updated the MEME installation guide         - Removed unused documents  ---   **MEME version 4.8.1 -- February 6, 2012**   - **New Features and Enhancements**   - `CentriMo`     - Added method to create custom Encapsulated Postscript       files of the motif probability graph.   - `MEME-ChIP`     - Added SpaMo to the list of programs that MEME-ChIP       runs. - **Bug fixes**   - Fixed a typo which was breaking the "Upload your own     database" feature on Tomtom.  ---   **MEME version 4.8.0 -- January 16, 2012**   - **New Features and Enhancements**   - `CentriMo`     - Added HTML output with lots of interactive features       allowing selecting, sorting and filtering of which motifs to       display and plot the site-probability curves for.     - Added `CentriMo` web submission form.     - `CentriMo` now runs as part of `MEME-ChIP`.     - Can now use motif matches to the given strand only if the       `-norc` switch is given. Useful with RNA sequences.     - Removed `centrimo-plots` program as the new       HTML output replaces and improves on its purpose.   - `DREME`     - Can now look for motifs on the given strand only if the       `-norc switch is given. This can be useful with       RNA sequences.`     - Using given strand only now available as an option on the web       submission form.   - `MCAST`     - Added support for scanning genome scale sequences.     - Added q-values for match scores.     - Revised HTML output to include block diagrams of matches.   - `SpaMo`     - SpaMo now normalizes the p-values reported for particular       motif spacings (bins) only for the number of spacings (bins)       tested. Previously, SpaMo reported p-values normalized for       both the number of spacings and the number of motifs tested.     - SpaMo now prints E-values for secondary motifs, which is the       lowest p-value of any spacing (bin) times the number of secondary       motifs.     - Added -evalue switch to specify largest E-value allowed for       secondary motifs.   - Other     - Improved memory efficiency for reading FASTA sequences in sequence.py. - **Bug fixes**   - Fixed segmentation fault in FIMO when option     `--bgfile motif-file` is used.   - Fixed png generation code for custom motif logos.  ---   **MEME version 4.7.0 -- September 22, 2011**   - **New Features and Enhancements**   - DREME can be accessed as a webservice directly which     allows use of the discriminative motif discovery.   - DREME now produces XML and HTML outputs as well as text.     These new outputs are accepted where MEME motifs are     accepted.   - New command-line program     CentriMo and the associated     CentriMo Plots for     determining which motifs are centrally enriched in ChIP-seq     output.   - Completely rewrote motif loading code. The main user     visible difference is that the minimal MEME format is a     lot more forgiving of spacing and MAST can now accept the     minimal MEME format without any PSSM section.   - Output fromm all tools nows lists prefered citation.   - Updated and re-formated list of publications. - **Bug fixes**   - Occasionally MEME would produce HTML files which could not     be used as a motif input to other programs. The new motif     parser should be able to read these files and hence fix this     problem.   - SpaMo background is now calculated from the sequences as     stated in the paper.   - The website menu used to use the full URL which     unfortunately resulted in people bookmarking specific versions     of the MEME suite rather than the URL for the latest version.     The website menu has been corrected to use only relative     URLs.   - Links should now be created to the error messages when     a webservice fails. This used to work but some setting changed     the way redirects are buffered.   - Added compiler/linker directives to set stack size for     Cygwin to 9MB. Cygwin defaults to a 2MB stack, which is too     small.   - MAST was not handling the `-remcorr` switch.     When a motif was removed because it was too correlated, the     indices of the other motifs could be incorrect. Fixed. - **Known issues**   - DREME does not yet support single strand scanning. It is     planned but wasn't ready for this release.   - Uploading extremely large sequence sets to MEME-ChIP can     cause a HTTP timeout and/or failure of the CGI script.  ---   **MEME version 4.6.1 -- March 21, 2011**   - **New Features and Enhancements**   - `MEME-ChIP` command line tool --      MEME-ChIP was released in 4.6.0 as a web service but it did not     have a command line equalivent. The webservice has been rewritten     as a command line tool.   - `TOMTOM` --       The default TOMTOM scoring algorithm now considers all columns in the     query motif, not just the aligned columns. This reduces the     number of spurious alignments including uninformative columns.     The older scoring system is available via the     `-incomplete-scores` option. - **Bug fixes**   - `MEME-ChIP` --      - MEME-ChIP has been rewritten to avoid infinite loops that       could occur when the webservice was passed parameters including       colons.     - The webservice now updates its output with the       completion of each sub-program so if it is teminated by Opal       for running too long the already completed output is still avaliable.     - Error reporting has been improved with the result that       MEME-ChIP should not be able to fail mysteriously as it has       on occasion been doing.   - FIMO     - The `--norc` option was being ignored.       This has been fixed.   - Motiph     - The `--seed` option has been added to set the       seed for the random number generator. - **Known issues**   - MEME-ChIP when given very large sequence sets will not complete before the Opal webservice time limit.   - Uploading extremely large sequence sets to MEME-ChIP can cause a HTTP timeout and/or failure of the CGI script.  ---   **MEME version 4.6.0 -- December 24, 2010**   - **New Features and Enhancements**   - `MEME-ChIP` web service --      - **Motif Analysis of Large DNA Datasets**        The MEME-ChIP service allows submission of a FASTA file of DNA sequences       of unlimited size and with minimal parameter settings (only the traditional       MEME web form settings) and runs it through a variety of motif finding and       analysis tools.     - `dreme` --        added `dreme` and integrated it into the MEME-ChIP into web service.     - `AME` --        added `ame` and integrated it into the MEME-ChIP into web service. This       is a cut-down version of the `ame` previously described, only supporting one mode       of motif enrichment analysis.     - `fasta-dinucleotide-shuffle` --        added `scripts/fasta-dinucleotide-shuffle` and integrated it into the MEME-ChIP into web service.       Thanks to Peter Clote for allowing use of his dinucleotide shuffle code.     - `fasta-center` --        added `fasta-center` and integrated it into the MEME-ChIP into web service.   - `SpaMo` beta release --      The web interface and documentation is currently rather limited but in the interests of     getting this out for review this is a preview edition of the **Spaced Motif analysis tool**.   - `FIMO` --       The `FIMO` HTML output now explicitly labels the strand,     rather than indicating the reverse strand by having start     > than `stop`.     The HTML output also now includes the matched sequence.     The GFF output is now GFF 3.   **MEME version 4.5.0 -- September 6, 2010**   - **New Features and Enhancements**   - `Tomtom` --      - **Search multiple databases**        Tomtom can now search more than one motif database.       To keep the ability to have web links for each motif       they were moved into the MEME motif format itself.       See the documentation.     - **Improvements in submission form**        Tomtom's submission form has many new enhancements.       Motifs can now be specified in 3 formats: IUPAC motifs,       frequency matrices, and MEME format files.       Go try it.     - **Improvements in output format**        Tomtom now outputs an XML file which it converts into       the HTML output. The HTML output can dynamically       draw the motif logos when the images aren't available       on web browsers supporting HTML5. Additional improvements       include a summary table at the top giving direct links to the       top 20 matches for each motif. See the       sample.   - `FIMO` --      The output for FIMO now includes a     wiggle track format file.   - MEME file format --      The MEME file format now includes an optional URL for each motif. This is used     to create the links in the Tomtom output. All the XXXX2meme scripts have been     retrofitted to take this URL parameter.   - `ama-qvalues` --      New script. See the documentation.   - `fasta-subsample` --      New script for extracting a random sampling of the sequences     in a FASTA file. This is especially useful for ChIP-seq     peak datasets to be input to MEME. Using this script, a     FASTA file containing a subset of the ChIP-seq peak sequences     (or any other FASTA file) can be created. The total     number of sequences should be less than 1000 (preferably less     than 500), and the total sequence length should be less     than a few hundred thousand. MEME typically takes about 20 minutes     per motif with files of 100,000 characters (DNA, both strands, ZOOPS model),     and scales quadratically in the total sequence length (so a file of     200,000 characters will take four times as long.)     This new script can also output the remaining sequences (in a seperate     file) for use in cross-validation. See the documentation.   - `meme2meme` --      New script. See the documentation.   - `rna2meme` --      New script. See the documentation.  ---   **MEME version 4.4.0 -- April 23, 2010**   - **New Features and Enhancements**    - `MCAST` --      MCAST     now been integrated into the MEME Suite web applications.     MCAST is a tool for searching for     statistically significant clusters of motifs in DNA sequences.     MCAST was previously supported on a separate web site.   - `MEME`      - **Discriminative motif discovery**          The MEME web server now supports discriminative       motif discovery. The user may now provide a set       of "negative sequences" in addition to the normal       "positive sequences", and MEME will look for       motifs that are overrepresented in the positive sequences       relative to the negative sequences.     - **Improvements in output format**          MEME's html output (sample) has been reorganised to       make important information more accessible as well as to make it aesthetically pleasing. Some of the improvements       include:        - A preview of the motifs found, including their reverse complement for DNA, in miniature at the top of the page.       - Ability to view full sized reverse-complemented DNA logos.       - Highlighting of all output for a sequence with a single click.       - New block diagrams that graphically show the strength (and DNA strand) of motifs,         and that scale with the size of the browser window.       - Support for the new web standard HTML5. This allows the motif logos to be viewed without the images         on supporting web browsers (Firefox, Chrome, Safari). Unfortunately Opera has inadequate text support for canvas         (the new HTML5 element required for this feat) and Internet Explorer currently doesn't support canvas at all.         It is anticipated that Internet Explorer 9 will support this feature.   - `MAST` --      - **Improvements in output format**     MAST's html output (sample) has been reorganised to     make important information more accessible as well as to make it aesthetically pleasing. Some of the improvements     include:      - Everything is now in one place - no longer do you have to search to find the associated annotated sequence for that       high scoring sequence, as with one click the hidden information is displayed directly below. Additionally if DNA       strands are scored separately then the sequence only appears in the list once based on the better scoring strand.     - New block diagrams - incorporating all the improvements in the new MEME block diagrams such as graphical display of       motif strength, scaling with the browser window and the DNA strand. As an added bonus separately scored strands       are visible on the same diagram.     - Interactive annotated sequence - a new range selector allows you to view the sequence around the motif hits of your       choice, directly relatable to the block diagram. For separate DNA strand scoring only one strand is visible at a time.     - Now in colour - the annotated sequence for motif hits now have letters colour coded using the same colour scheme       as MEME motifs.     - MAST now outputs xml in a similar fashion to MEME. This has resulted in differences to how you would invoke MAST       from the command line. Additionally it no longer has the script wrapper.     - MAST now uses an xml style sheet to create its html output.   - `mast2txt` --      Created program mast2txt for outputting the old text format for MAST. Note that there are some     problems with backwards compatibility in separate scoring mode. The use of the text format is no longer recommended.   - `psp-gen` --      added `psp-gen` and integrated PSP generation into web service.   - `MHMM` --      Fixed bug in `--order` option.     The option was handling motifs with strand identifiers incorrectly.   - `TOMTOM` --       Added `-no-ssc` switch to fix problem with     motifs with few sites having empty logos; this switch is now     the default for the TOMTOM website.   - `GOMO` --      Improved gomo html format.     Added highlighting of the most specific GO terms in     the html output. This is activated by the switch --dag     which expects a GO DAG file.   - `gomo_highlight` --      Added a program to post process gomo xml files to     identify the go terms which are implied by other     more specific go terms to allow the specific terms     to be highlighted in the html output. This is used     by gomo when the --dag switch is specified.   - `obo2dag` --      Added a tool for generating a GO DAG file from a     Gene Ontology OBO file.   - `GO DAG format` --      Documented the new GO DAG file format and improved GOMO     documentation as well as the command line overview.   - `GLAM2` --       The output now prints a regular expression in addition to the logo     for each motif found.   - `MEME motif format` --      Fixed errors in motif format description     `doc/meme-format.html` and example files.   - `meme-io` --      Updated meme-io to accept the new MEME html format.  --- |
